# Association of worrier trait with the risk of Parkinson's disease: a longitudinal study based on 457,180 UK Biobank participants

**DOI:** 10.3389/fpsyg.2025.1440199

**Published:** 2025-03-25

**Authors:** Rui Li, Yitong Ling, Ao Pan, Rui Cao, Jun Lyu, Wei Bi

**Affiliations:** ^1^Department of Neurology, The First Affiliated Hospital of Jinan University, Guangzhou, China; ^2^Department of Clinical Research, The First Affiliated Hospital of Jinan University, Guangzhou, China

**Keywords:** worry, worrier trait, mental health, Parkinson's disease, cohort study

## Abstract

**Objective:**

To explore the potential association between the trait of being a worrier and the likelihood of developing Parkinson's disease (PD).

**Background:**

While extensive research has explored the link between PD and disorders such as depression and anxiety, limited research has been conducted on the association between worry and PD.

**Methods:**

This prospective cohort study utilized data obtained from the UK Biobank, with baseline assessments conducted between 2006 and 2010 and follow-up until July 16, 2023. Multivariable Cox proportional hazards regression analysis was carried out to evaluate the link between worrier trait and the development of PD. Adjustments were made for demographic factors, smoking, PD polygenetic risk scores, alcohol consumption, body mass index, physical activity, stroke, diabetes, hypertension, heart attack, and psychiatric history. Subgroup and sensitivity analyses were additionally conducted to validate the reliability of the outcomes.

**Results:**

Over a mean follow-up period of 13.5 years, 3123 participants (0.68%) out of 457,180 participants [median (IQR) age, 58.00 (50.00, 63.00) years; 54.3% female] developed PD. The incidence of PD was positively linked to worry (log-rank test, *P* < 0.001). Furthermore, worriers demonstrated a heightened risk of developing PD [hazard ratio (HR) 1.32, 95% CI 1.23–1.42]. Importantly, this link persisted even following adjustments for covariates (fully adjusted model HR 1.27, 95% CI 1.18–1.37). Additionally, when cases within the initial 5 years of follow-up were excluded, the significance of the association persisted (HR: 1.28, 95% CI 1.18–1.38). In subgroup analyses categorized by age, early-onset PD (age < 60 years) showed a stronger association than late-onset PD (age ≥ 60 years; early-onset PD HR 1.32, 95% CI 0.86–2.03; late-onset PD HR 1.13, 95% CI 1.05–1.22).

**Conclusion:**

These findings suggest that the worrier trait is consistently associated with a higher risk of developing PD, particularly among young individuals, highlighting the importance of mental wellness.

## Introduction

Parkinson's disease (PD) ranks as the second highly prevalent neurodegenerative disease following Alzheimer's disease and is witnessing rapid growth, as evidenced by the 6 million people afflicted worldwide currently (Dorsey et al., 2018; Feigin et al., 2019). Epidemiological research has firmly established that behavioral and environmental elements may be involved in the onset and progression of the disease. For example, risk factors for PD include consumption of dairy products, pesticide exposure, a history of cancer, and traumatic brain injury. On the contrary, factors such as smoking, heightened serum urate concentrations, caffeine consumption, and physical activity have been found to decrease the risk of PD (Ascherio and Schwarzschild, 2016).

PD is an intricate neurodegenerative condition characterized by bradykinesia, resting tremor, and rigidity. Additionally, it may manifest with autonomic dysfunction, cognitive impairments, and mood disturbances (Armstrong and Okun, 2020). The etiology of PD is multifaceted and complex. Recently, there has been growing interest in exploring the potential influence of psychological factors on its development. Specifically, various psychiatric conditions and psychosocial issues, such as bipolar disorder, schizophrenia, and loneliness, have been suggested to increase the risk of PD (Faustino et al., 2020; Huang et al., 2019; Kuusimäki et al., 2021; Terracciano et al., 2023). Among these, depression and anxiety have been extensively studied as risk factors for PD (Ishihara-Paul et al., 2008; Schuurman et al., 2002; Chelminski and Zimmerman, 2003; Wang et al., 2018; Nilsson et al., 2001). A meta-analysis of 11 studies confirms that depression is significantly associated with an increased risk of PD (Wang et al., 2018). Another study has revealed a positive correlation between mood disorders and the risk of developing PD (Noyce et al., 2012).

In summary, previous research has predominantly focused on the relationship between severe mental issues and PD, overlooking the association between more common psychological traits and PD. As a common human experience, the worry trait may be an important, yet understudied, psychological factor contributing to PD risk.

Worry is defined as a chain of negatively charged thoughts and images that are relatively uncontrollable. It represents an attempt to mentally address issues with uncertain outcomes, which may involve one or more negative consequences (Borkovec et al., 1983). Those who worry often engage in mental rehearsals of potential future events and experiences they perceive as dangerous or undesirable while simultaneously seeking strategies to avoid them (Mathews, 1990). Previous theoretical and empirical research has solidified the notion that worry, particularly its repetitive nature, can serve as a predictor of anxiety and depression (Gustavson et al., 2018; Fresco et al., 2002; Segerstrom et al., 2000). Newman et al. further supported this perspective, emphasizing that persistent, frequent, and uncontrollable worry can pose challenges and is linked to various psychiatric disorders, encompassing depression and anxiety disorders (McEvoy et al., 2013). Worry has been found to be equally linked to symptoms of depression, anxiety, and stress (Newman et al., 2013). And existing research has largely focused on anxiety and depression as potential risk factors for PD. Relatively less is known about the association between worry and PD. Elucidating this association could enhance our comprehension of PD's risk factors and underlying mechanisms. Addressing this gap is crucial for early diagnosis and intervention strategies for PD.

As mentioned above, psychiatric disorders are linked to a heightened risk of PD. Nonetheless, to our knowledge, there is currently little evidence to indicate whether mild emotional distress, specifically worry, poses a risk for developing PD. Hence, this study aims to explore the relationship between the trait of worry and the risk of PD over an extended follow-up period using a population-based cohort from the UK Biobank project. The research also seeks to test whether this association is moderated by other risk factors. Finally, this association is further tested in subgroups and sensitivity analyses.

## Methods

### Study population

The UK Biobank (UKB) is an ongoing population-based prospective study with participants exceeding 500,000 (Sudlow et al., 2015). From 2006 to 2010, individuals were invited to one of 22 assessment centers throughout the UK for baseline information collection. At the baseline visit, participants were asked to complete a touchscreen questionnaire, providing information regarding sociodemographic characteristics, lifestyle factors, and health status. Subsequent assessments are scheduled at intervals of 2 to 3 years. Approval for the UKB study was granted by the North West Multi-Center Research Ethics Committee (REC reference: 11/NW/03820). The provision of written informed consent was considered essential for participation, aligning with the principles of the Declaration of Helsinki (Sudlow et al., 2015).

**Figure 1:**
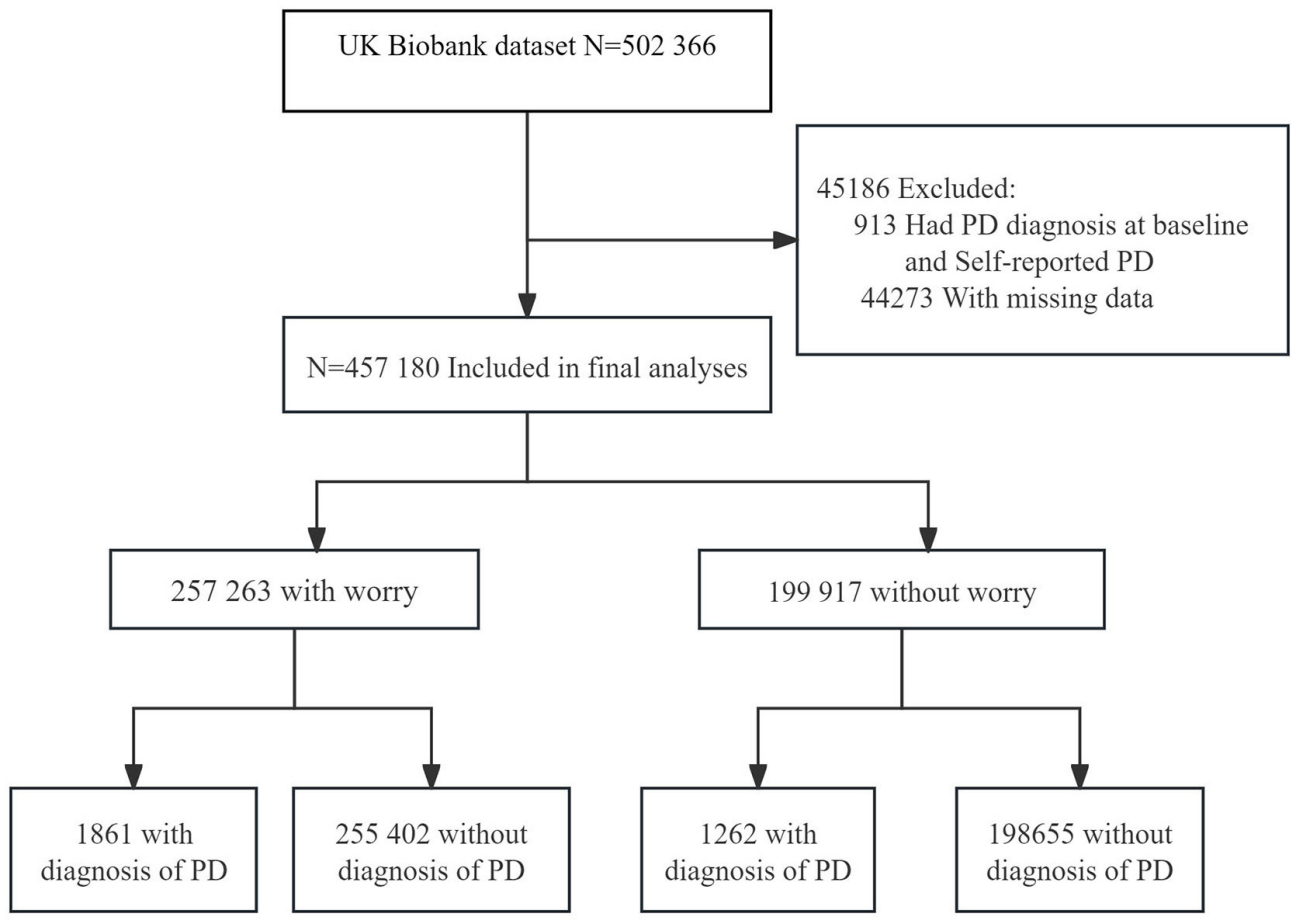
Flowchart of included participants in the study.

In this retrospective cohort investigation, the initial inclusion criteria encompassed 502,366 UKB participants. To ensure consistency and reliability in the study population and minimize biases, we excluded individuals as follows: (1) individuals with a PD diagnosis at baseline, (2) self-reported PD, to reduce the risk of misdiagnosis and improve the validity of the study findings, (3) those who did not respond to specific baseline inquiries, as well as participants who selected “prefer not to answer” or “do not know” were excluded to ensure that the analysis was based on a complete and reliable data set. Approximately 0.08% of the total sample size had incomplete data that was excluded from the analysis. Ultimately, the study cohort consisted of 457,180 individuals (see Figure 1).

### Evaluation of worry

The mental health of the participants was assessed using an electronic questionnaire upon entry to the assessment center. Our primary focus was on evaluating the factor of worry, which was measured by the question, “Are you a worrier?.” Responses were coded as 0 for no and 1 for yes.

### Outcome assessment

Cases of PD were identified through algorithmically defined outcomes integrating self-reported medical conditions, hospital admissions, and death registries. The date of PD diagnosis was extracted from the UKB data field labeled “Date of Parkinson's Disease report.” Self-reported PD instances and cases with a PD diagnosis predating baseline were excluded from the analysis of incident PD. The follow-up duration was calculated from the date of the worry assessment to the date of PD diagnosis, date of fatality, or the end of follow-up (July 16, 2023), whichever occurred first.

### Covariates

Based on previous research evidence (Noyce et al., 2012; Ye et al., 2021), we included the following covariates in our study to comprehensively analyze the factors influencing the relationship between worry and PD. The additional covariates were as follows: age (continuous), sex (female/male), alcohol consumption (never/previous/current), smoking (never/previous/current), and education (college/university degree, other). Body mass index (BMI, continuous) was calculated utilizing standing height and weight values assessed at the assessment center. The Townsend deprivation index (TDI) comprises a composite score derived from four primary variables: unemployment, overcrowded households, non-car ownership, and non-home ownership. The index has been validated and applied in population studies within the UK, where higher scores correlate with greater levels of deprivation (Ye et al., 2021). Physical activity was assessed by asking participants, “How many days did you walk for at least 10 min at a time?”

Conditions such as hypertension and diabetes have been associated with an increased risk of PD (Simonet et al., 2022). So we took into account some comorbidities that may act as confounding factors, influencing the relationship between worry and PD risk. Medical history data were acquired via a standard self-administered questionnaire, including inquiries about diabetes (yes/no), stroke (yes/no), hypertension (yes/no), and heart attack (yes/no) history. To assess any instance of prior psychiatric services, the item, “Have you ever seen a psychiatrist for nerves, anxiety, tension, or depression?” (yes/no) was utilized. The polygenic risk score (PRS) is a useful predictive tool that quantifies a patient's cumulative genetic risk by compiling genome-wide significant variants, helping to identify high-risk patients (Dehestani et al., 2021). Standard PD PRS were available from the UKB genomic data, which had been extensively described in prior sources (Thompson et al., 2022).

### Statistical analyses

Baseline attributes were separately summarized for individuals with and without worry and PD. Categorical variables were expressed as percentages, while continuous variables were presented as means with standard deviations (SDs) or medians with interquartile ranges (IQRs). A preliminary assessment of the potential link between worry and the risk of developing PD was conducted using cumulative risk curves and the log-rank test.

Next, multivariate Cox proportional hazards regression models were utilized for estimating the association of worry with the likelihood of incident PD, with adjustment for confounding factors (reported using hazard ratios, HRs, and 95% confidence intervals, CIs). By creating multiple models that include different sets of covariates, we aimed to isolate the specific effect of worry on PD risk. This approach allowed us to observe how the relationship between worry and PD risk changed when accounting for various demographic, lifestyle, medical, and psychological factors. It provided a comprehensive analysis of the relationship between worry and PD risk. Research has shown that age and sex are the main factors in the incidence of PD (Willis et al., 2022; Wooten et al., 2004); therefore, four multivariate-adjusted models were developed in this study, each incorporating age and sex. Among these, model 1 incorporated sex and age. Model 2 encompassed all of the factors in model 1 along with college education, TDI scores, BMI, smoking, alcohol and physical activity. Model 3 incorporated the factors from model 2 along with diabetes, stroke, hypertension, heart attack. Model 4 encompassed the factors from model 3 plus PD PRSs and history of psychiatric services, was all adjusted model.

Lastly, to affirm the robustness of the acquired data, additional subgroup and sensitivity analyses were carried out. It is widely acknowledged that PD is more common in men and older adults; therefore, the above analyses were also conducted in subgroups stratified by sex, age ( ≤ 60 years and > 60 years), and other covariates to identify potential effect modifications. Furthermore, subjects who developed PD with a follow-up of < 5 years underwent exclusion from the investigation to minimize the influence of reverse causality on the model. Statistical analyses were executed utilizing R software (v 4.2.2). *P* < 0.05 was deemed as a statistically significant value.

## Results

### Basic attributes of the control and PD groups

After excluding individuals with previously diagnosed PD, self-reported PD, and missing baseline data, 457,180 participants, ranging from 38 to 73 years in age [median (IQR) age, 58.00 (50.00, 63.00) years], were selected for participation in the investigation. Figure 1 exhibits the flow chart illustrating the study selection strategy.

During the maximum 16 years of follow-up [mean (SD), 13.5 (2.1) years, 6,171,930 person-years], a total of 3,123 participants developed PD, resulting in an incidence rate of 3,123 per 6,171,930 person-years (51/100,000 person-years). Among them, 1,861 cases were from the worrier group (incidence rate, 53/100,000 person-years), and 1,262 cases occurred in the non-worrier group (incidence rate, 46/100,000 person-years). Individuals who reported being worriers exhibited a higher likelihood of being female, having hypertension, or having sought psychiatrist help for nerves, anxiety, tension, or depression. Additionally, these individuals were noted to have a lower likelihood of attaining a university or college degree. These differences are delineated in Table 1.

**Table 1:** Baseline characteristics of study participants by worrier.

	**Not worrier**	**Worrier**	**P**
N	199,917	257,263	
Age [median (IQR)]	58.00 [50.00, 63.00]	58.00 [50.00, 63.00]	< 0.001
**Sex (%)**			< 0.001
Female	90,010 (45.0)	158,332 (61.5)	
Male	109,907 (55.0)	98,931 (38.5)	
College education (%)	68,872 (34.5)	81,688 (31.8)	< 0.001
**Smoking (%)**			< 0.001
Never	108,431(54.2)	141,848 (55.1)	
Previous	70,014 (35.0)	89,370 (34.7)	
Current	21,472 (10.7)	26,045 (10.1)	
**Alcohol (%)**			< 0.001
Never	8,035 (4.0)	10,984 (4.3)	
Previous	6,379 (3.2)	9,584 (3.7)	
Current	185,503 (92.8)	236,695 (92.0)	
BMI [median (IQR)]	27.03 [24.49, 30.12]	26.44 [23.84, 29.60]	< 0.001
TDI [median (IQR)]	−2.21 [−3.68, 0.38]	−2.18 [−3.67, 0.46]	< 0.001
Physical activity [median (IQR)]	6.00 [4.00, 7.00]	6.00 [4.00, 7.00]	< 0.001
PD-PRS [mean (SD)]	−0.14 (1.02)	−0.14 (1.02)	0.237
Have seen a psychiatrist (%)	12,562 (6.3)	39,562 (15.4)	< 0.001
Diabetes (%)	11,053 (5.5)	12,189 (4.7)	< 0.001
Stroke (%)	2,835 (1.4)	3,788 (1.5)	0.13
Hypertension (%)	5,0216 (25.1)	72,256 (28.1)	< 0.001
Heart attack (%)	4,716 (2.4)	5,577 (2.2)	< 0.001

BMI, body mass index; TDI, Townsend deprivation index; PD-PRS, Parkinson disease polygenic risk scores; IQR, interquartile range; SD, standard deviation.

Compared to the entire study population, individuals with PD were noted to be older [median age 64.0 years, IQR (60.00, 67.00)], more often male, and exhibited a history of smoking and drinking. Additionally, they were less educated, had a higher genetic risk for PD and BMI, and were more prone to comorbidities such as diabetes, stroke, hypertension, and heart attack. Notably, they were more likely to consult a psychiatrist for mental health issues (see Supplementary Table S2).

### Association between worry and PD risk

**Figure 2:**
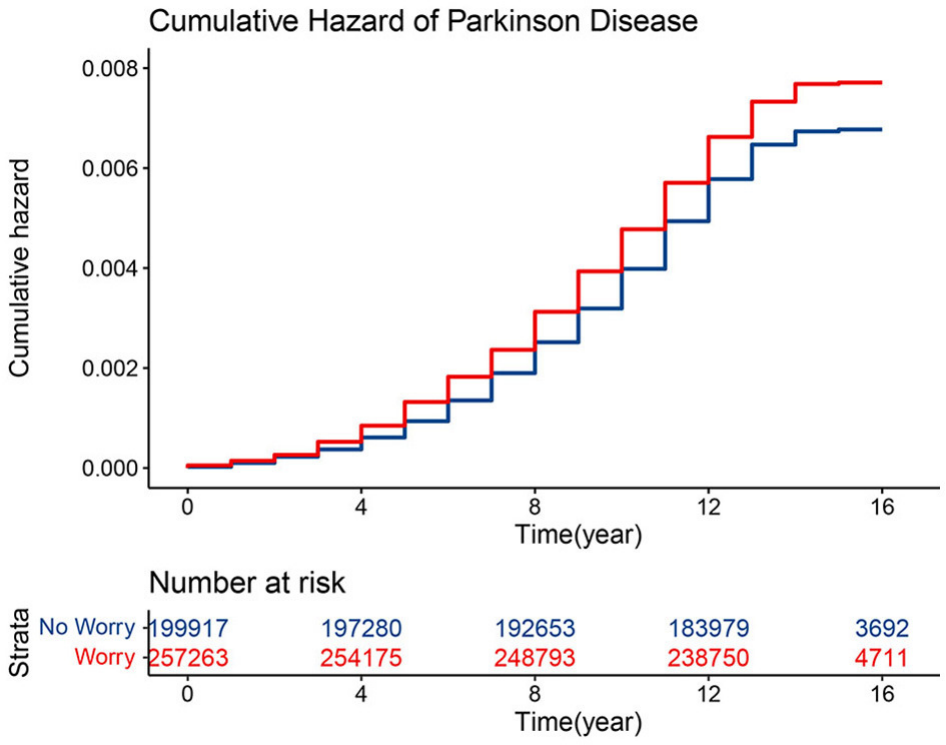
Cumulative incidence of PD among groups with and without worry.

A Kaplan-Meier cumulative risk curve revealed a greater risk of developing PD in the group that reported worrying than in the group that did not. Subsequently, the statistically significant differences between the two groups were assessed using log-rank tests (*P* < 0.001). It was observed that the differences in cumulative risk between the two groups became more pronounced as the follow-up time increased (see Figure 2).

**Figure 3:**
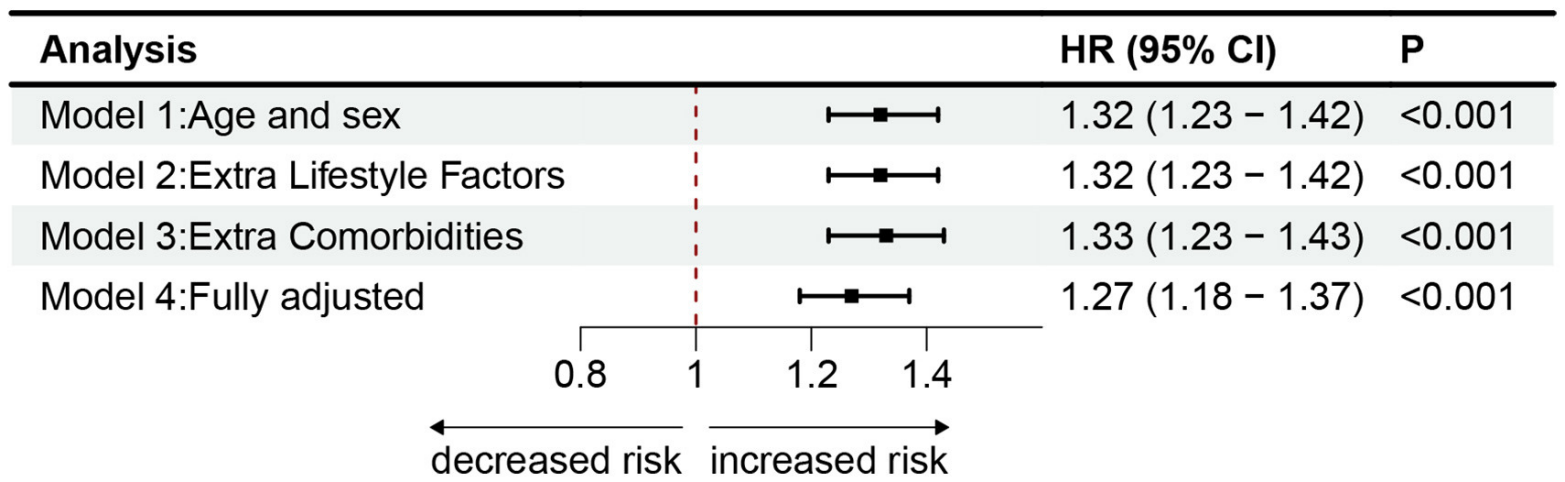
The cox proportional risk models estimating the hazard ratio of PD. Association of worry with incident PD in models with different sets of covariates and the fully adjusted model. Model 1 incorporated sex and age. Model 2 included the factors from Model 1, as well as college education, TDI scores, BMI, smoking, alcohol consumption, and physical activity. Model 3 added diabetes, stroke, hypertension, and heart attack to the factors in Model 2. Model 4 further included PD PRSs and history of psychiatric services, representing the fully adjusted model. Markers indicate hazard ratios (HRs), with horizontal lines representing 95% CIs. The vertical line indicates a reference value of 1.

The main results of the four Cox proportional hazards regression models created to assess the association between worry and the risk of PD are presented in Supplementary Table S3. Each model incorporated sex and age as covariates. In model 1, worry was linked to a heightened risk of PD (HR 1.32, 95% CI 1.23–1.42). In model 2, we further adjusted for lifestyle (HR 1.32, 95% CI 1.23–1.42). In model 3, additional adjustments were made for comorbidities such as diabetes, stroke, hypertension, heart attack (HR 1.33, 95% CI 1.23–1.43). Notably, the outcomes implied that these confounding factors did not weaken the associations between worry and PD risk. These associations were attenuated but remained significant when adjusting for the covariates of having sought help from a psychiatrist for depression or anxiety and PD PRSs in the fully adjusted model 4 (HR 1.27, 95% CI 1.18–1.37). Figure 3 shows the forest plot of all model results.

### Subgroups and sensitivity analyses

**Figure 4:**
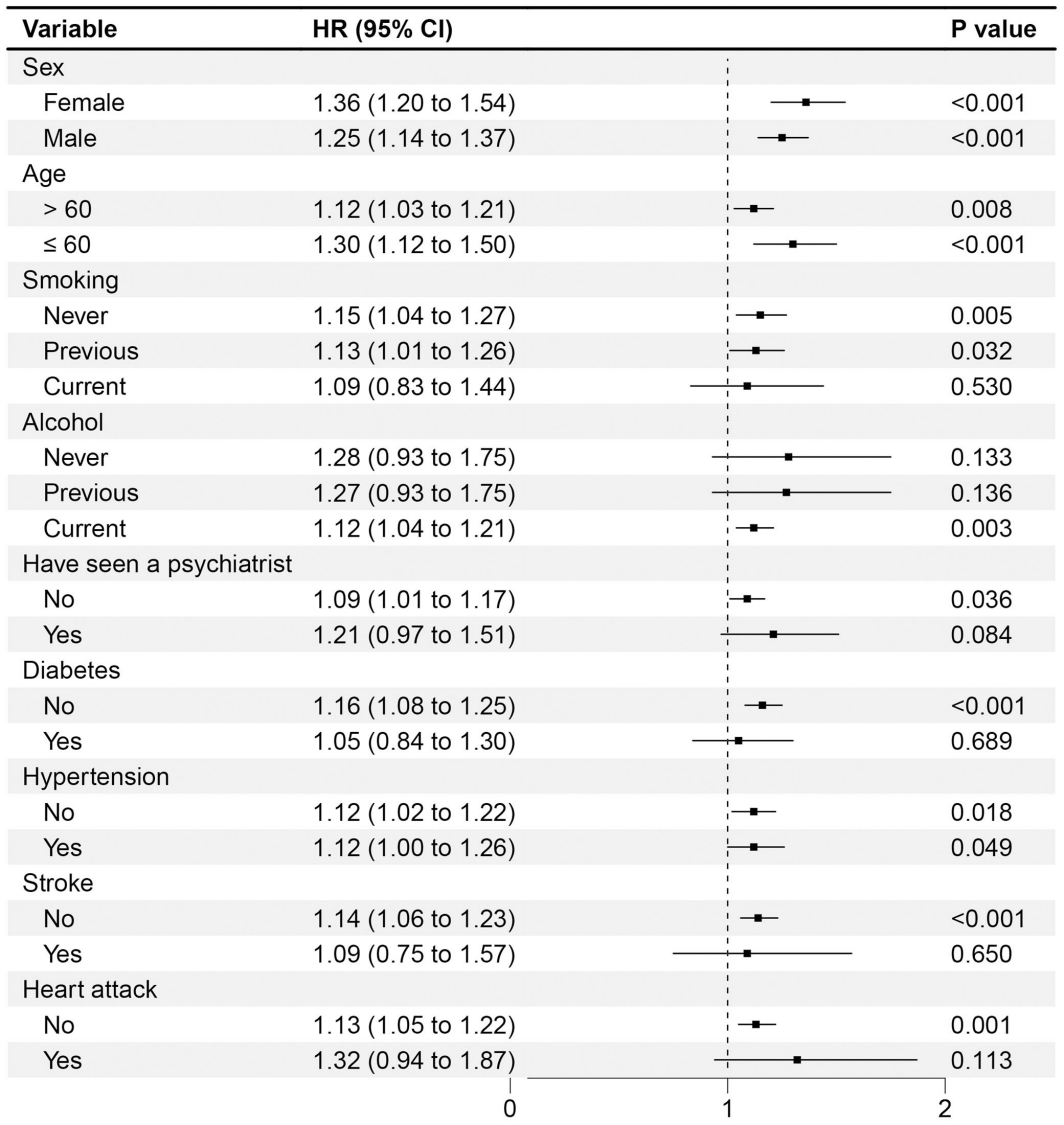
Association of worry and the risk of PD with different subgroups stratified by potential risk factors. The multivariable model was adjusted for age, sex, smoking, alcohol, physical activity, body mass index, education, TDI, PD-PRS, diabetes, stroke, hypertension, heart attack and ever seen a psychiatrist. The vertical line indicates a reference value of 1.

Notably in Figure 4, the subgroups and sensitivity analyses all supported the main findings mentioned above. However, there are some noteworthy observations. Firstly, in the analysis of subgroups by age, a more pronounced association between worry and PD was observed in the younger subgroup (HR 1.30, 95% CI 1.12–1.50) compared to the subgroup aged over 60 years (HR 1.12, 95% CI 1.03–1.21). Secondly, in analyses stratified by sex, worry was noted to be related to a heightened risk of PD in women (HR 1.36, 95% CI 1.20–1.54); however, this association was attenuated in men (HR 1.25, 95% CI 1.14–1.37). We performed multiple comparisons correction to the subgroup analyses using false discovery rate (FDR) correction (Benjamini and Hochberg), and key findings remained robust (see Supplementary Table S5). Lastly, compared with the crude regression analyses, worry remained a significant risk predictor for incident PD following the exclusion of cases diagnosed during the initial 5 or 7 years of follow-up in the multivariate regression analysis (HR 1.28, 95% CI 1.18–1.38, HR 1.23, 95% CI 1.13–1.34).

## Discussion

This population-based cohort study, with an average follow-up period of 13.5 years, primarily explored the impact of worry on the likelihood of developing PD. It attempted to elucidate the relationship between these two elements. The acquired data depicted a significant impact of worry on PD, revealing that individuals who experience worry are at a greater risk of developing PD. Additionally, adjusted models indicated that confounding factors exhibited minimal influence on this association. Subgroup and sensitivity analyses also consistently demonstrated that worry is linked to an elevated risk of PD, further supporting our findings. Notably, the subgroup analyses indicated that individuals under 60 years of age or with a follow-up time of 5 years or less displayed a stronger association with PD risk compared to those over 60 years. Furthermore, a surprising discovery emerged from this large-scale study: the incidence of worry within the population was unexpectedly high, exceeding half of the total population. This finding underscores the significant influence of worry on PD risk and the need for further investigation into this relationship.

The majority of prior research has primarily dealt with the various non-motor symptoms of PD. Psychiatric issues, such as depression, anxiety, and sleep disturbances, are frequently observed during the course of PD (Global Parkinson's Disease Survey (GPDS) Steering Committee, 2002; Forsaa et al., 2008). However, recent investigations have implied that psychiatric problems may precede the incidence of PD, potentially elevating its risk. A retrospective study examining the link between depression and PD risk reported that subjects with depression exhibited a heightened risk of developing PD (Shen et al., 2013), a result that is congruent with the outcomes of a prior meta-analysis (Wang et al., 2018). Another study, which evaluated the clinical predictive significance of affective disorders in the development of PD among women aged 65 years and older, revealed a doubled risk of PD among this population (Beydoun et al., 2022). Consistent with prior research, our study suggests that individuals who frequently worry are at a high risk of developing PD, even if they do not fulfill the clinical criteria for psychiatric disorders.

The potential link between worry and PD appears to be multifaceted. On one hand, worry may serve as a risk factor for PD, possibly mediated by depression and anxiety. Worry is not limited to Generalized Anxiety Disorder but is also present in other emotional disorders, such as anxiety, depression, and obsessive-compulsive disorder, which suggests a coexistence of these conditions and shared risk factors for PD (Chelminski and Zimmerman, 2003). Worry is often considered a manifestation of the personality trait neuroticism. High neuroticism may adversely affect brain health and raise the likelihood of neurodegenerative diseases (Terracciano et al., 2021). On a more mechanistic level, monoamine neurotransmission and chronic inflammation are hypothesized to serve as bridges connecting depression and PD (Remy et al., 2005). On the other hand, another crucial mechanism involves worry potentially activating chronic inflammation in the body as a chronic stressor, thereby elevating the risk of developing PD. Recently, an increasing body of research has supported the involvement of immune dysregulation and inflammation in the pathogenesis of PD (Lauritsen and Romero-Ramos, 2023; Cossu et al., 2023; Fu et al., 2023). There is even a hypothesis suggesting that the aging immune system, in conjunction with complex gene-environment interactions, contributes to the development and progression of PD (Tansey et al., 2022). Given that chronic stressors are linked to the dysregulation of the hypothalamic-pituitary-adrenal axis and higher levels of systemic inflammatory markers such as CRP (Marsland et al., 2017), it is plausible that worry may activate the immune system, leading to chronic inflammation in the body, which could subsequently contribute to the occurrence of PD.

Another possible interpretation is that worry, as a psychological condition, may manifest in the preclinical stages of PD. Studies have found that individuals with PD often experience psychological symptoms such as depression, anxiety, and tension during the prodromal phase. These symptoms are observed 3 to 6 years before the onset of the disease (Tolosa et al., 2007). However, it should be noted that worry is a common human experience, and its association with PD remains significant even after accounting for psychological symptoms. And we used the longitudinal data, where worry was assessed before the onset of PD, provides stronger evidence for a temporal sequence leading from worry to PD. Furthermore, even when excluding cases diagnosed within the initial 5 years or seven years of follow-up, the association between worry and the incidence of PD remains significant. Therefore, it is unlikely that the link between worry and PD is merely due to coincidental or overlapping preclinical symptoms. We suggest that future studies explore the temporal relationship between worry and PD more comprehensively to better understand the potential causal pathway.

Our findings suggest that individuals below the age of 60 who experience worry have a stronger association with the risk of PD compared to those older than 60, which aligns with previous research (Yoon et al., 2024). There are several possible interpretations of these findings. Firstly, younger individuals are more likely to experience higher levels of stress and worry, which could potentially lead to increased neuroinflammatory processes and oxidative stress, both known contributors to PD pathogenesis. Additionally, worry may interact with certain environmental factors or an individual's lifestyle and habits, potentially increasing the risk of early-onset PD. For example, an unhealthy diet has been associated with PD risk (Tresserra-Rimbau et al., 2023). Furthermore, worriers may be more prone to seeking medical attention and receiving an earlier diagnosis of PD. Finally, as individuals age, other risk factors such as genetic factors, long-term exposure to certain environmental factors, or increased comorbidities may exert a more significant role in the onset of PD. This could diminish the predictive effect of worry on PD risk.

In the subgroup analysis, another interesting finding was that the association between worry and PD was more pronounced among females. A previous study reported that women with anxiety have shown double the increased PD risk (Beydoun et al., 2022). However, there is little elaboration on the mechanisms related to this aspect. Women may be more susceptible to the effects of stress and emotional factors on their health. Gender differences in hormonal profiles, particularly fluctuations in estrogen levels, could play a role in modulating the relationship between worry and PD risk. Additional research is warranted to replicate these findings in other cohorts and delve into the underlying mechanisms governing this relationship.

Our research has several strengths. Firstly, while prior studies have focused on the influence of mental illnesses on neurodegenerative diseases, there has been little emphasis on specifically exploring the impact of worry on PD. Secondly, this study used data from the large population-based UKB cohort, which provided a significant advantage in terms of a large sample size, long follow-up period, and detailed information on potential confounders. Additionally, we unexpectedly found that worry was associated with a higher risk of early-onset PD, despite the fact that the prevalence of early-onset PD is low compared to late-onset PD. Furthermore, the relationship between worry and PD appears to be particularly robust among females, indicating a potential gender-specific susceptibility to the effects of worry on PD risk.

Nevertheless, several limitations should be acknowledged in this study. Firstly, although prior research has demonstrated the generalizability of exposure-outcome associations within the UKB (Batty et al., 2020), our study did not establish causal inferences. It is known that PD presents non-motor symptoms many years before motor symptoms manifest. Those worriers may already have non-motor symptoms of PD including depression or anxiety while scoring “worrier.” Although the results continued to show a correlation between worry and PD by additional and sensitivity analyses. The possibility of reverse causality cannot be dismissed. Future research can focus on incorporating biomarkers of PD pathology, such as cerebrospinal fluid markers or imaging biomarkers, which could help identify individuals at risk of developing PD before motor symptoms emerge, allowing for a more accurate assessment of the relationship between worry and PD. Secondly, several covariates, as well as the years of initial PD diagnoses, were based on self-reported data, which might introduce response bias. Thirdly, our study focused solely on the association between worry and PD, without exploring the impact of the frequency, severity, or specific nature and content of worrying thoughts on PD risk. Lastly, the study only employed a self-reporting approach to gather information on worry in individuals. Although using a single question allows for consistency in data collection and high response rates across a large cohort study, it is important to note that, the single-item measure may introduce greater error variance and may not capture the complexity of worry.

Future research can use more standardized and specialized psychological assessment instruments to investigate this relationship accurately. These assessments could unveil the underlying mechanisms that link psychological states to neurodegenerative processes, offering insights into potential intervention points (Zarotti et al., 2021). Mild cognitive impairment is one of the common non-motor symptoms in PD patients (Yu and Wu, 2022). Studies could explore whether interventions that target worry could mitigate cognitive decline and, by extension, the risk of developing PD.

## Conclusion

In conclusion, our findings revealed that individuals who are prone to worrying were at a higher risk of developing PD, even after adjusting for genetic predispositions and established social, behavioral, and clinical risk factors. Notably, this association appeared to be stronger among younger individuals. Given the high prevalence of worry in the general population, it is crucial to prioritize mental health in today's stressful environment. Doing so could profoundly impact both the prevention and management of PD.

## Data Availability

The data analyzed in this study is subject to the following licenses/restrictions: the data are obtainable from UK Biobank and have been utilized under the terms of a license agreement. Requests to access these datasets should be directed to https://www.ukbiobank.ac.uk/.
