# Supplementary material for: Association of worrier trait with the risk of Parkinson's disease: a longitudinal study based on 457,180 UK Biobank participants
Source: Front Psychol. 2025 Mar 25;16:1440199. doi: 10.3389/fpsyg.2025.1440199 (PMC11995634; doi:10.3389/fpsyg.2025.1440199)
Supplement: Supplementary file 1 [file Data_Sheet_1.pdf]

Supplementary Material

Association of worrier trait with the risk of Parkinson's disease: a longitudinal study based on 457,180 UK Biobank participants

Rui Li[1,†], Yitong Ling[1,†], Ao Pan[1], Rui Cao[1], Jun Lyu,[2,*], Wei Bi, [1,*]

†First Author
Rui Li: lyrui929@stu.jnu.edu.cn
Yitong Ling: lingyitong@stu2022.jnu.edu.cn

*** Correspondence:**
Wei Bi: tbwneurodoc@jnu.edu.cn
Jun Lyu: lyujun2020@jnu.edu.cn

This supplementary material has been kindly provided to give readers additional information about the study.

Supplemental table S1. UK Biobank Field codes for all variables
Supplemental table S2. Baseline characteristics of study participants by incident PD
Supplemental Table S3. Hazard Ratios of the Association of Worry with Incident PD Accounting for Different Sets of Covariates and Fully Adjusted Model
Supplemental Table S4. Subgroups analyses stratified by potential risk factors
Supplemental Table S5. Comparison of raw and adjusted P-Values across subgroups using Benjamini-Hochberg Corrections

Supplemental table S1 UK Biobank Field codes for all variables

variables	Field ID
Date of attending assessment centre	53
Age at recruitment	21022
Sex	31
Smoking	20116
Alcohol	20117
BMI	21001
PD_PRS	26260
College.education	6138
TDI	22189
Physical.activity	864
Have.seen.a.psychiatrist	2100
Diabetes	2443
Stroke	6150
Hypertension	6150
Heart attack	6150
Have you ever seen a psychiatrist for nerves, anxiety, tension or depression?	2100
Worrier	1980
Deathtime	40000
PD-date	42032
PD-Source	42033

BMI,body mass index;TDI, Townsend deprivation index; PD-PRS, Parkinson disease polygenic risk scores; PD,Parkinson disease.

Supplemental Table S3 Hazard Ratios of the Association of Worry with Incident PD Accounting for Different Sets of Covariates and Fully Adjusted Model

	Model1		Model2		Model3		Model4	
	HR	95%CI	HR	95%CI	HR	95%CI	HR	95%CI
**Age**	1.15	1.14 - 1.16	1.15	1.14 - 1.16	1.15	1.14 - 1.16	1.15	1.14 - 1.15
**Sex**	2.02	1.88 - 2.18	2.07	1.93 - 2.24	2.02	1.87 - 2.18	2.03	1.88 - 2.19
**Worry**	1.32	1.23 - 1.42	1.32	1.23 - 1.42	1.33	1.23 - 1.43	1.27	1.18 - 1.37
**College education**			0.98	0.91 - 1.06	0.99	0.91 - 1.07	0.96	0.89 - 1.04
**TDI**			1.01	1.00 - 1.03	1.01	1.00 - 1.02	1.01	0.99 - 1.02
**BMI**			1.01	1.00-1.02	1.00	0.99 - 1.01	1.40	1.35 - 1.45
**Smoking previous**			0.93	0.86 - 1.00	0.92	0.86 - 1.00	0.92	0.85 - 0.99
**Smoking current**			0.74	0.64 - 0.85	0.73	0.63 - 0.85	0.71	0.62 - 0.83
**Alcohol previous**			1.23	0.99 - 1.53	1.22	0.99 - 1.52	1.19	0.96 - 1.48
**Alcohol current**			0.76	0.65 - 0.89	0.78	0.66 - 0.92	0.79	0.68 - 0.93
**Physical activity**			0.99	0.97 - 1.01	0.99	0.98 - 1.01	1.00	0.98 - 1.01
**Diabetes**					1.49	1.32 - 1.68	1.49	1.32 - 1.68
**Stroke**					1.66	1.38 - 2.00	1.66	1.38 - 2.00
**Hypertension**					1.06	0.98 - 1.14	1.06	0.98 - 1.14
**Heart attack**					1.00	0.84 - 1.2	1.00	0.84 - 1.19
**PD-PRS**							1.00	0.99 - 1.01
**Have seen a psychiatrist**							1.48	1.34 - 1.64

Supplemental Table S4 Subgroups analyses stratified by potential risk factors

Variable	Count	Percent	HR	Lower	Upper	P value	P for interaction
Overall	457180	100	1.14	1.06	1.22	<0.001	
Sex							0.279
Female	248342	54.3	1.36	1.2	1.54	<0.001	
Male	208838	45.7	1.25	1.14	1.37	<0.001	
Age							0.080
> 60	175087	38.3	1.12	1.03	1.21	0.008	
<= 60	282093	61.7	1.30	1.12	1.50	<0.001	
Smoking							0.918
Never	250279	54.7	1.15	1.04	1.27	0.005	
Previous	159384	34.9	1.13	1.01	1.26	0.032	
Current	47517	10.4	1.09	0.83	1.44	0.53	
Alcohol							0.564
Never	19019	4.2	1.28	0.93	1.75	0.133	
Previous	15963	3.5	1.27	0.93	1.75	0.136	
Current	422198	92.3	1.12	1.04	1.21	0.003	
Have seen a psychiatrist							0.346
No	405056	88.6	1.09	1.01	1.17	0.036	
Yes	52124	11.4	1.21	0.97	1.51	0.084	
Diabetes							0.364
No	433938	94.9	1.16	1.08	1.25	<0.001	
Yes	23242	5.1	1.05	0.84	1.3	0.689	
Hypertension							0.92
No	334708	73.2	1.12	1.02	1.22	0.018	
Yes	122472	26.8	1.12	1	1.26	0.049	
Stroke							0.808
No	450557	98.6	1.14	1.06	1.23	<0.001	
Yes	6623	1.4	1.09	0.75	1.57	0.65	
Heart attack							0.389
No	446887	97.7	1.13	1.05	1.22	0.001	
Yes	10293	2.3	1.32	0.94	1.87	0.113	

Supplemental Table S5 Comparison of raw and adjusted P-Values across subgroups using Benjamini-Hochberg Corrections

Variable	Count	Percent	HR	Lower	Upper	P value	Adjusted P value
Sex							
Female	248342	54.3	1.36	1.2	1.54	<0.001	0.003
Male	208838	45.7	1.25	1.14	1.37	<0.001	0.003
Age							
> 60	175087	38.3	1.12	1.03	1.21	0.008	0.018
<= 60	282093	61.7	1.3	1.12	1.5	<0.001	0.003
Smoking							
Never	250279	54.7	1.15	1.04	1.27	0.005	0.013
Previous	159384	34.9	1.13	1.01	1.26	0.032	0.058
Current	47517	10.4	1.09	0.83	1.44	0.53	0.589
Alcohol							
Never	19019	4.2	1.28	0.93	1.75	0.133	0.160
Previous	15963	3.5	1.27	0.93	1.75	0.136	0.160
Current	422198	92.3	1.12	1.04	1.21	0.003	0.009
Have seen a psychiatrist							
No	405056	88.6	1.09	1.01	1.17	0.036	0.060
Yes	52124	11.4	1.21	0.97	1.51	0.084	0.120
Diabetes							
No	433938	94.9	1.16	1.08	1.25	<0.001	0.003
Yes	23242	5.10	1.05	0.84	1.30	0.689	0.689
Hypertension							
No	334708	73.2	1.12	1.02	1.22	0.018	0.036
Yes	122472	26.8	1.12	1.00	1.26	0.049	0.075
Stroke							
No	450557	98.6	1.14	1.06	1.23	<0.001	0.003
Yes	6623	1.4	1.09	0.75	1.57	0.65	0.684
Heart.attack							
No	446887	97.7	1.13	1.05	1.22	0.001	0.003
Yes	10293	2.3	1.32	0.94	1.87	0.113	0.151
